# Supplementary material for: The multi-drug resistant organisms infections decrease during the antimicrobial stewardship era in cirrhotic patients: An Italian cohort study
Source: PLoS One. 2023 Feb 16;18(2):e0281813. doi: 10.1371/journal.pone.0281813 (PMC9934314; doi:10.1371/journal.pone.0281813)

**Table 1.2 (additional): Infections, bacterascites and asymptomatic bacteriuria during the study period.**

| **Infections recorded during the follow up, n (%)** | 101 (%) |
| --- | --- |
| Sepsis, n (%) | 25 (24.7) |
| Pneumonia, n (%) | 20 (19.8) |
| SBP, n (%) | 18 (17.8) |
| Site unknown, n (%) | 18 (17.8) |
| UTI, n (%) | 12 (11.9) |
| Cholangitis, n (%) | 3 (3) |
| SSTI, n (%) | 3 (3) |
| Gastroenteritis, n (%) | 2 (2) |
| **Bacterascites, n** | 15 |
| **Asymptomatic bacteriuria, n** | 25 |

*SBP, spontaneous bacterial peritonitis; UTI, urinary tract infection; SSTI, skin and soft tissues infection.*

| Sepsis |  |
| --- | --- |
| Bloodstream | MDR *Pseudomonas* |
|  | MR coagulase negative Staphylococci |
|  | VRE |
|  | ESBL *K. pneumoniae* |
|  | MRSA |
| Pneumonia |  |
| Bloodstream | MRSA |
|  | ESBLO *K. pneumoniae* |
| BAL | MDR *Pseudomonas* |
| UTI |  |
| Urine | MDR *Proteus* |
|  | MDR *Pseudomonas* |
|  | ESBL *K. pneumoniae* |
| Bloodstream | VRE |
| Peritonitis |  |
| Ascites | MR coagulase negative Staphylococci |
|  | ESBL *E. coli* |
|  | VRE |

**Table 1.3 (additional): specific isolated bacteria considering infection and source of isolation**

*MDR, multi-drug resistant; MR, methicillin-resistant; VRE, vancomycin-resistant Enterococci; ESBL,* *extended spectrum beta lactamase; MRSA, methicillin-resistant Staphylococcus Aureus; BAL, bronchoalveolar lavage; UTI, urinary tract infection.*

**Table 4.1 (additional): Multivariate analysis with outcome death as dependent variable**

| **Variable** | **Multivariate** | |
| --- | --- | --- |
|  | **OR (95% CI)** | **p-value** |
| Age | 1.04 (1.00 – 1.07) | **0.031** |
| Male sex | 0.88 (0.44 – 1.77) | 0.727 |
| Diabetes | 0.44 (0.21 – 0.92) | **0.029** |
| Hypertension | 0.82 (0.42 – 1.62) | 0.582 |
| CKD | 1.73 (0.61 – 4.85) | 0.300 |
| PH | 1.08 (0.53 – 2.20) | 0.835 |
| Ascites | 0.69 (0.35 – 1.36) | 0.284 |
| Infections | 3.30 (1.63 – 6.70) | **<0.001** |

*CKD, chronic kidney disease; PH, portal hypertension.*

**Flow-chart SAVE program**


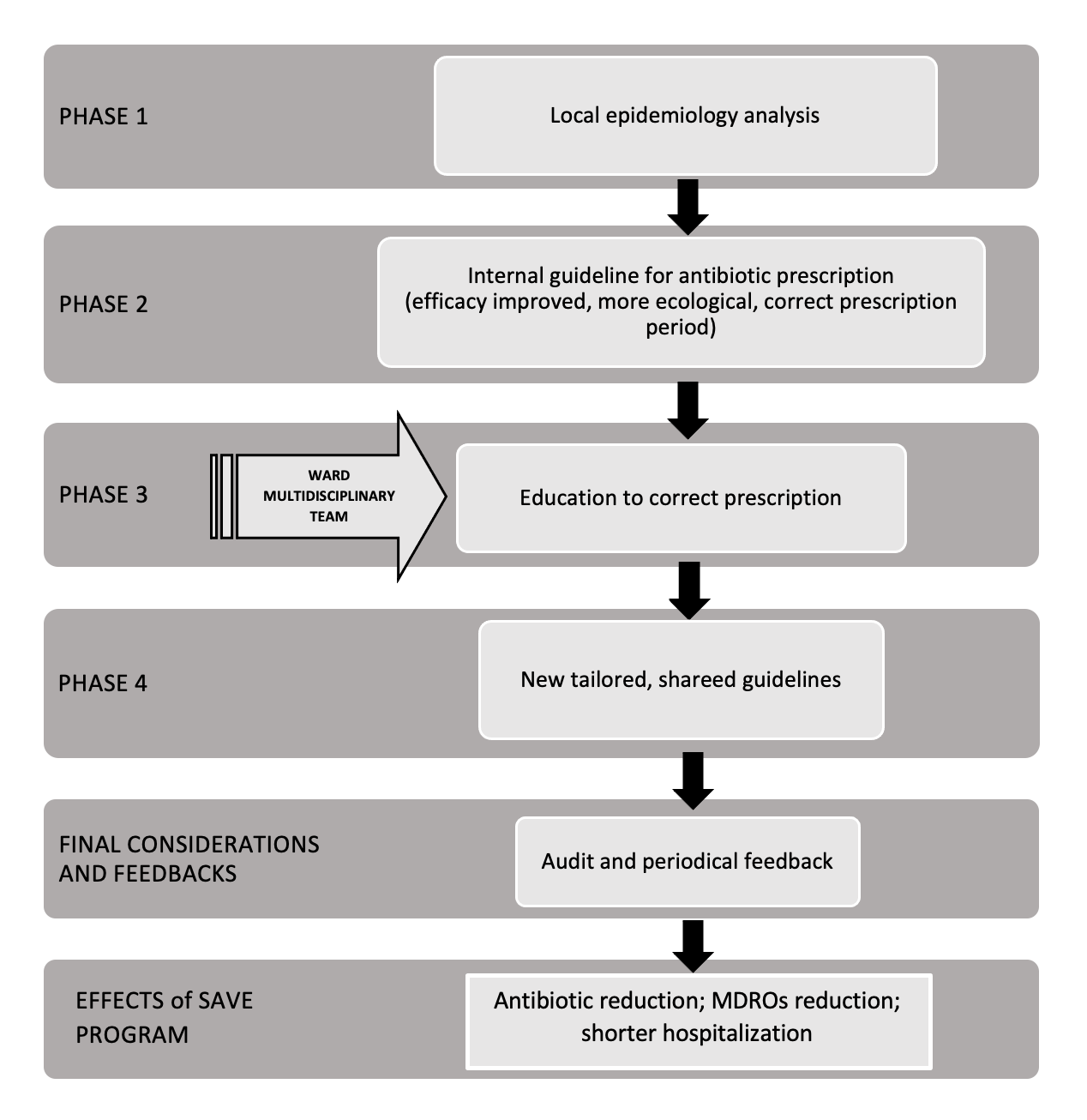

Supplement: S1 File — (DOCX) [file pone.0281813.s001.docx]
